# GDF-11 promotes human trophoblast cell invasion by increasing ID2-mediated MMP2 expression

**DOI:** 10.1186/s12964-022-00899-z

**Published:** 2022-06-15

**Authors:** Ze Wu, Lanlan Fang, Sizhu Yang, Yibo Gao, Zhen Wang, Qingxue Meng, Xuan Dang, Ying-Pu Sun, Jung-Chien Cheng

**Affiliations:** Henan Key Laboratory of Reproduction and Genetics, Center for Reproductive Medicine, The First Affiliated Hospital of Zhengzhou University, 40 Daxue Road, Zhengzhou, 450052 Henan China

**Keywords:** GDF-11, BMP-11, MMP2, Trophoblast cells, Invasion

## Abstract

**Background:**

Growth differentiation factor-11 (GDF-11), also known as bone morphogenetic protein-11, belongs to the transforming growth factor-beta superfamily. GDF-11 was first identified as an important regulator during embryonic development. Increasing evidence has demonstrated that GDF-11 regulates the development of various organs and its aberrant expressions are associated with the risk of cardiovascular diseases and cancers. Extravillous trophoblast (EVT) cells invasion is a critical event for placenta development and needs to be finely regulated. However, to date, the biological function of GDF-11 in the human EVT cells remains unknown.

**Methods:**

HTR-8/SVneo, a human EVT cell line, and primary cultures of human EVT cells were used to examine the effect of GDF-11 on matrix metalloproteinase 2 (MMP2) expression. Matrigel-coated transwell invasion assay was used to examine cell invasiveness. A series of in vitro experiments were applied to explore the underlying mechanisms that mediate the effect of GDF-11 on MMP2 expression and cell invasion.

**Results:**

Treatment with GDF-11 stimulates MMP2 expression, in the HTR-8/SVneo and primary human EVT cells. Using a pharmacological inhibitor and siRNA-mediated knockdown approaches, our results demonstrated that the stimulatory effect of GDF-11 on MMP2 expression was mediated by the ALK4/5-SMAD2/3 signaling pathways. In addition, the expression of inhibitor of DNA-binding protein 2 (ID2) was upregulated by GDF-11 and that was required for the GDF-11-stimulated MMP2 expression and EVT cell invasion.

**Conclusions:**

These findings discover a new biological function and underlying molecular mechanisms of GDF-11 in the regulation of human EVT cell invasion.

**Video Abstract**

**Supplementary Information:**

The online version contains supplementary material available at 10.1186/s12964-022-00899-z.

## Background

The transforming growth factor-beta (TGF-β) is a large superfamily that consists of many structurally related members including TGF-βs, growth differentiation factors (GDFs), bone morphogenetic proteins (BMPs), activins, inhibins, and anti-Mullerian hormone [1]. The TGF-β superfamily affects many physiological and pathological events by regulating various cellular functions that are in a cell- and/or context-specific manner [2]. Placentation is a process modulated by a complex interaction between the placental trophoblast cells and the endometrium. Placental development is marked by extravillous trophoblast (EVT) cells invading the decidua and spiral arteries, replacing the cells of the vessel wall and creating a high-flow low-resistance vessel that ensures a continuous blood supply to the placenta throughout pregnancy. Aberrant EVT cell invasion is associated with different placental diseases such as preeclampsia, intrauterine growth restriction, miscarriage, and hydatidiform mole [3, 4]. To date, it is known that several members of the TGF-β superfamily are expressed in the human placenta and regulate EVT cell invasion in an autocrine and/or paracrine manner [5–7].

GDF-11, also known as BMP-11, is a member of the TGF-β superfamily and plays important role in the regulation of anterior/posterior axial patterning during embryonic development [8–10]. Deletion of the *Gdf11* gene in mice results in perinatal lethality. Although the precise cause of death remains unknown, *Gdf11*^*–/–*^ mice died within 24 h after birth [11]. Mutations in human *GDF11* and its extracellular antagonist, *FST*, genes are associated with the orofacial clefts [12]. Various in vivo studies in rodents have demonstrated the postnatal functions of GDF-11 in skeletal muscle, heart, brain, and bone and suggested potential therapeutic implications in those organs-related diseases [13]. In humans, the GDF-11 expression can be detected in nearly all organs including the placenta [14]. Aberrant expressions of GDF-11 are associated with the pathogenesis of cardiovascular, neurological, skeletal muscle, and age-related diseases as well as the risk of cancers [15, 16].

Similar to other TGF-β superfamily members, GDF-11 activates intracellular signaling by binding to two activin type-II receptors, ActRIIA and ActRIIB, and three type-I receptors, activin receptor-like kinase 4 (ALK4), ALK5, and ALK7 that belong to serine and threonine kinase receptors. Upon ligand binding, activated receptors phosphorylate and activate the canonical SMAD2/3 or SMAD1/5/8 signaling pathways and non-canonical ERK1/2, p38, and JNK signaling pathways [13, 16]. We have shown that TGF-β1 inhibits invasiveness in primary human EVT cells and an immortalized EVT cell line, HTR-8/SVneo [17–19]. In contrast, activins and GDF-8 stimulate human EVT cell invasion [20, 21]. These studies indicate that different TGF-β superfamily members have distinct effects on the regulation of human EVT cell invasion. GDF-11 and GDF-8 share 89% sequence identity in their mature form and are believed to have similar biological functions because the same membrane receptors and intracellular signaling pathways are used and activated, respectively. However, in some contexts, GDF-11 and GDF-8 exert different biological functions [13, 22]. Matrix metalloproteinase 2 (MMP2) and MMP9 mediated remodeling and degradation/activation of the extracellular matrix play an essential role in the EVT cell invasion [23]. Our recent study shows that GDF-8 stimulates human EVT cell invasion by upregulating the expression of MMP2 [21]. However, to date, the biological function of GDF-11 in human EVT cells remains unknown, and whether GDF-11 has a similar effect to GDF-8 in the regulation of EVT cell invasion is unclear. Therefore, the present study was designed to examine the effect of GDF-11 on human EVT cell invasion and to explore related underlying molecular mechanisms.

## Materials and methods

### Antibodies and reagents

The MMP2 (#40,994), phospho-SMAD2 (#3108), phospho-SMAD3 (#9520), SMAD2 (#3103), SMAD3 (#9523), SMAD4 (#38454), phospho-SMAD1/5/8 (#13820), SMAD1 (#6944), and ID2 (#3431) antibodies were obtained from Cell Signaling Technology. The α-tubulin (#sc-23948) and vimentin (#sc-6260) antibodies were obtained from Santa Cruz Biotechnology. The cytokeratin-7 antibody (#MAB3554) was obtained from Millipore. The HLA-G antibody (#11-499) was obtained from EXBIO. The recombinant human GDF-11 and BMP4 were obtained from R&D systems. The SB431542 was obtained from Sigma.

### Cell culture and reagents

The HTR-8/SVneo cell line was obtained from American Type Culture Collection through an official distributor in China (Beijing Zhongyuan Limited). HTR-8/SVneo is an SV40 large T antigen immortalized first-trimester short-lived EVT cell line [24]. Cells were cultured in a humidified atmosphere containing 5% CO_2_ and 95% air at 37 °C in Dulbecco’s modified Eagle’s medium/nutrient mixture F-12 Ham medium (DMEM/F-12; Gibco) supplemented with 10% FBS (HyClone), 100 U/mL penicillin and 100 μg/mL streptomycin sulfate (Boster).

### Primary human EVT cell isolation and culture

The study received institutional approval and was carried out in accordance with the guidelines from the Zhengzhou University Research Ethics Board (#2020-KY-140). Human EVT cells were isolated from first-trimester (6–9 weeks of gestation) placental tissue explants as previously described [18, 19]. Briefly, chorionic villi were washed with a cold medium and mechanically minced into 1–2 mm fragments. Fragments of the chorionic villi were allowed to adhere for 2–3 days, after which any non-adherent material was removed. These tissue explants were further cultured for 10–14 days to allow EVT cell outgrowth, during which the culture medium was changed every 2 days. EVT cells were separated from the villous explants by brief trypsin digestion. Cells were plated in a 6-well or 12-well plate (2 × 10^4^ cell/cm^2^) without coating and cultured in a humidified atmosphere containing 5% CO_2_ and 95% air at 37 °C in Dulbecco’s modified Eagle’s medium/nutrient mixture F-12 Ham medium (DMEM/F-12) supplemented with 10% FBS, 100 U/mL penicillin, and 100 μg/mL streptomycin sulfate. Isolated primary EVT cells were characterized by the expressions of cytokeratin-7 and HLA-G. Primary EVT cells were not passaged. Individual primary cultures were composed of cells from one individual patient. Each experiment was repeated at least three times and each time used cells derived from different patients.

### Immunofluorescence staining

Cells were cultured on coverslips, fixed with cold methanol at − 20 °C, and then permeabilized with 0.1% Triton X-100 in phosphate-buffered saline (PBS). Cells were blocked with Dako Protein Block (Dako) for 1 h and incubated with antibodies diluted in Dako Protein Block. Alexa 488-labeled donkey anti-mouse was used as a secondary antibody. Cells were counterstained with DAPI, rinsed with PBS, mounted with Gelvatol, and examined using a Nikon Eclipse fluorescence microscope.

### Reverse transcription quantitative real-time PCR (RT-qPCR)

Total RNA was extracted with the RNeasy Plus Mini Kit (QIAGEN) according to the manufacturer’s instructions. RNA (1 μg) was reverse-transcribed into first-strand cDNA with the iScript Reverse Transcription Kit (Bio-Rad Laboratories). Each 20 μL qPCR reaction contained 1X SYBR Green PCR Master Mix (Applied Biosystems), 60 ng of cDNA, and 250 nM of each specific primer. The following primers were used: MMP2, 5′-TAC ACC AAG AAC TTC CGT CTG T-3′ (sense) and 5′-AAT GTC AGG AGA GGC CCC AT-3′ (antisense); MMP9, 5′-TTG ACA GCG ACA AGA AGT GG-3′ (sense) and 5′-CCC TCA GTG AAG CGG TAC AT-3′ (antisense); ALK4, 5′-TCT CTC CAC CTC AGG GTC TG-3′ (sense) and 5′-GCC ATA CTT CCC CAA ACC GA-3′ (antisense); ALK5, 5′-GTT AAG GCC AAA TAT CCC AAA CA-3′ (sense) and 5′-ATA ATT TTA GCC ATT ACT CTC AAG G-3′ (antisense); SMAD2, 5′-CCG AAA TGC CAC GGT AGA AA-3′ (sense) and 5′-GGG CTC TGC ACA AAG ATT GC-3′ (antisense); SMAD3, 5′-CCC CAG CAC ATA ATA ACT TGG-3′ (sense) and 5′-AGG AGA TGG AGC ACC AGA AG-3′ (antisense); SMAD4, 5′-TCC ACA GGA CAG AAG CCA TT-3′ (sense) and 5′-GTC ACT AAG GCA CCT GAC CC-3′ (antisense); ID2, 5′-CCC ACT ATT GTC AGC CTG CA-3′ (sense) and 5′-CTG CAA GGA CAG GAT GCT GA-3′ (antisense); and GAPDH, 5′-GAG TCA ACG GAT TTG GTC GT-3′ (sense) and 5′-GAC AAG CTT CCC GTT CTC AG-3′ (antisense). qPCR was performed on an Applied Biosystems QuantStudio 12 K Flex system equipped with 96-well optical reaction plates. The specificity of each assay was validated by melting curve analysis and agarose gel electrophoresis of the PCR products. All of the RT-qPCR experiments were run in triplicate, and a mean value was used to determine the mRNA levels. Water and mRNA without RT were used as negative controls. Relative quantification of the mRNA levels was performed using the comparative Ct method with GAPDH as the reference gene and using the formula 2^–∆∆Ct^.

### Western blot

Cells were lysed in cell lysis buffer (Cell Signaling Technology) supplemented with a protease inhibitor cocktail (Sigma). Equal amounts of protein were separated by SDS polyacrylamide gel electrophoresis and transferred onto PVDF membranes. After 1 h of blocking with 5% nonfat dry milk in Tris-buffered saline (TBS), the membranes were incubated overnight at 4 °C with primary antibodies diluted in 5% nonfat milk/TBS. Following primary antibody incubation, the membranes were incubated with appropriate HRP-conjugated secondary antibodies. Immunoreactive bands were detected using an enhanced chemiluminescent substrate (Bio-Rad Laboratories) and imaged with a ChemiDoc MP Imager (Bio-Rad Laboratories).

### Small interfering RNA (siRNA) transfection

To knock down endogenous ALK4, ALK5, SMAD2, SMAD3, SMAD4, ID2, or MMP2 cells were transfected with 50 nM ON-TARGETplus SMARTpool siRNA targeting a specific gene (Dharmacon) using Lipofectamine RNAiMAX (Invitrogen). The siCONTROL NON-TARGETING pool siRNA (Dharmacon) was used as the transfection control.

### Invasion assay

Transwell cell culture inserts (8 µm pore size, 24 wells, BD Biosciences) were coated with 1 mg/mL growth factor-reduced Matrigel (BD Biosciences). Cells (1 × 10^5^ cells/insert) in DMEM/F-12 medium supplemented with 0.1% FBS were incubated for 48 h against a gradient of 10% FBS. Non-invasive cells were removed with a cotton swab from the upper side of the membrane. Cells that penetrated the membrane were fixed with cold methanol, stained with crystal violet (0.5%, Sigma) for 30 min, and subsequently washed thoroughly with tap water. Each experiment was performed with triplicate inserts. In each insert, five microscopic fields were photographed under an optical microscope, and the cell number was counted manually.

### Statistical analysis

The results are presented as the mean ± SEM of at least three independent experiments. All statistical analyses were analyzed by PRISM software. Multiple comparisons were analyzed using one-way ANOVA followed by Tukey’s multiple comparison test. A significant difference was defined as *p* < 0.05. Values that are statistically different from one another (*p* < 0.05) are indicated by different letters. The values with any common letter are not significantly different.

## Results

### GDF-11 upregulates MMP2 but not MMP9 in human EVT cells

**Fig. 1:**
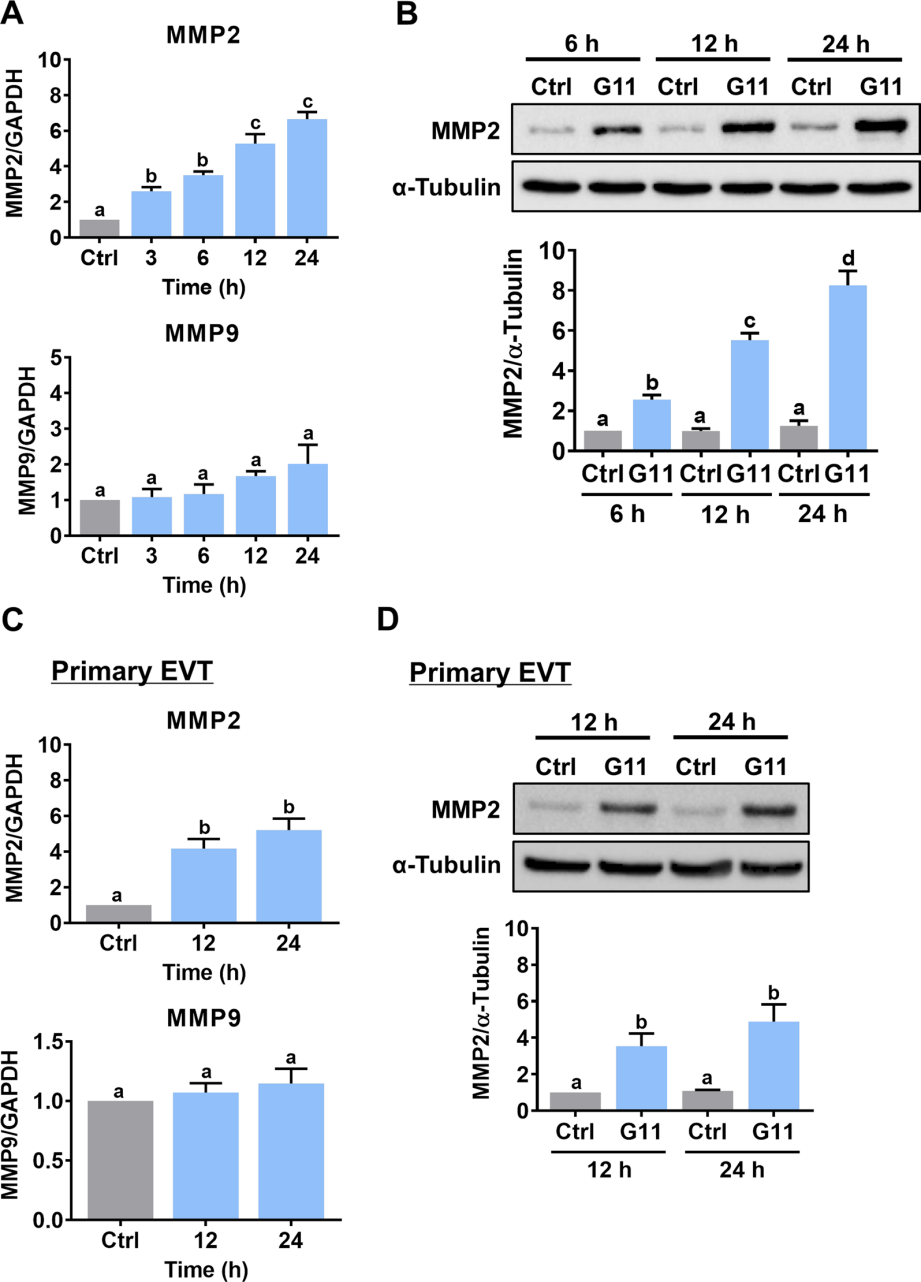
GDF-11 upregulates MMP2 but not MMP9 expression. **A**, HTR-8/SVneo cells were treated with 30 ng/mL GDF-11 for different periods, and the mRNA levels of MMP2 and MMP9 were examined by RT-qPCR. The level of MMP2 or MMP9 mRNA at each time point was normalized to the GAPDH mRNA level at the same time point. **B**, HTR-8/SVneo cells were treated with 30 ng/mL GDF-11 (G11) for 6, 12, and 24 h. The protein levels of MMP2 were examined by western blot. **C**, Primary human EVT cells were treated with 30 ng/mL GDF-11 for 12 and 24 h, and the mRNA levels of MMP2 and MMP9 were examined by RT-qPCR. The level of MMP2 or MMP9 mRNA at each time point was normalized to the GAPDH mRNA level at the same time point. **D**, Primary human EVT cells were treated with 30 ng/mL GDF-11 (G11) for 12 and 24 h. The protein levels of MMP2 were examined by western blot. The results are expressed as the mean ± SEM of at least three independent experiments. Multiple comparisons were analyzed using one-way ANOVA followed by Tukey’s multiple comparison test. Values that are statistically different from one another (*p* < 0.05) are indicated by different letters. The values with any common letter are not significantly different

The HTR-8/SVneo cell line was generated using human first trimester EVT cells infected with SV40 large T antigen and is the most commonly used cell model for studying the biological function of EVT cells [24]. Using this cell line makes the experiments more technically feasible, particularly for those involving gene manipulations. In women of reproductive age, the serum level of GDF-11 can reach 40 ng/mL [25]. To examine the effects of GDF-11 on MMP2 and MMP9 expressions, HTR-8/SVneo cells were treated with 30 ng/mL recombinant human GDF-11 for different periods. RT-qPCR results showed that GDF-11 upregulated MMP2 mRNA levels in a time-dependent manner. However, the mRNA levels of MMP9 were not affected by the treatment with GDF-11 (Fig. 1A). The effects of GDF-11 on MMP2 protein levels were examined by the western blot analysis. As shown in Fig. 1B, consistent with the RT-qPCR results, GDF-11 upregulated MMP2 protein levels in HTR-8/SVneo cells. To further confirm these results, primary EVT cells isolated from first-trimester chorionic villi were used. The characteristics of EVT cells were confirmed by the expressions of cytokeratin-7 and HLA-G (Additional file 1: Fig. S1). Similarly, treatment of GDF-11 upregulated MMP2 but not MMP9 mRNA levels in primary EVT cells (Fig. 1C). The stimulatory effect of GDF-11 on MMP2 protein levels was also observed by the western blot analysis (Fig. 1D).

### GDF-11 upregulates MMP2 expression through ALK4 and ALK5 in human EVT cells

**Fig. 2:**
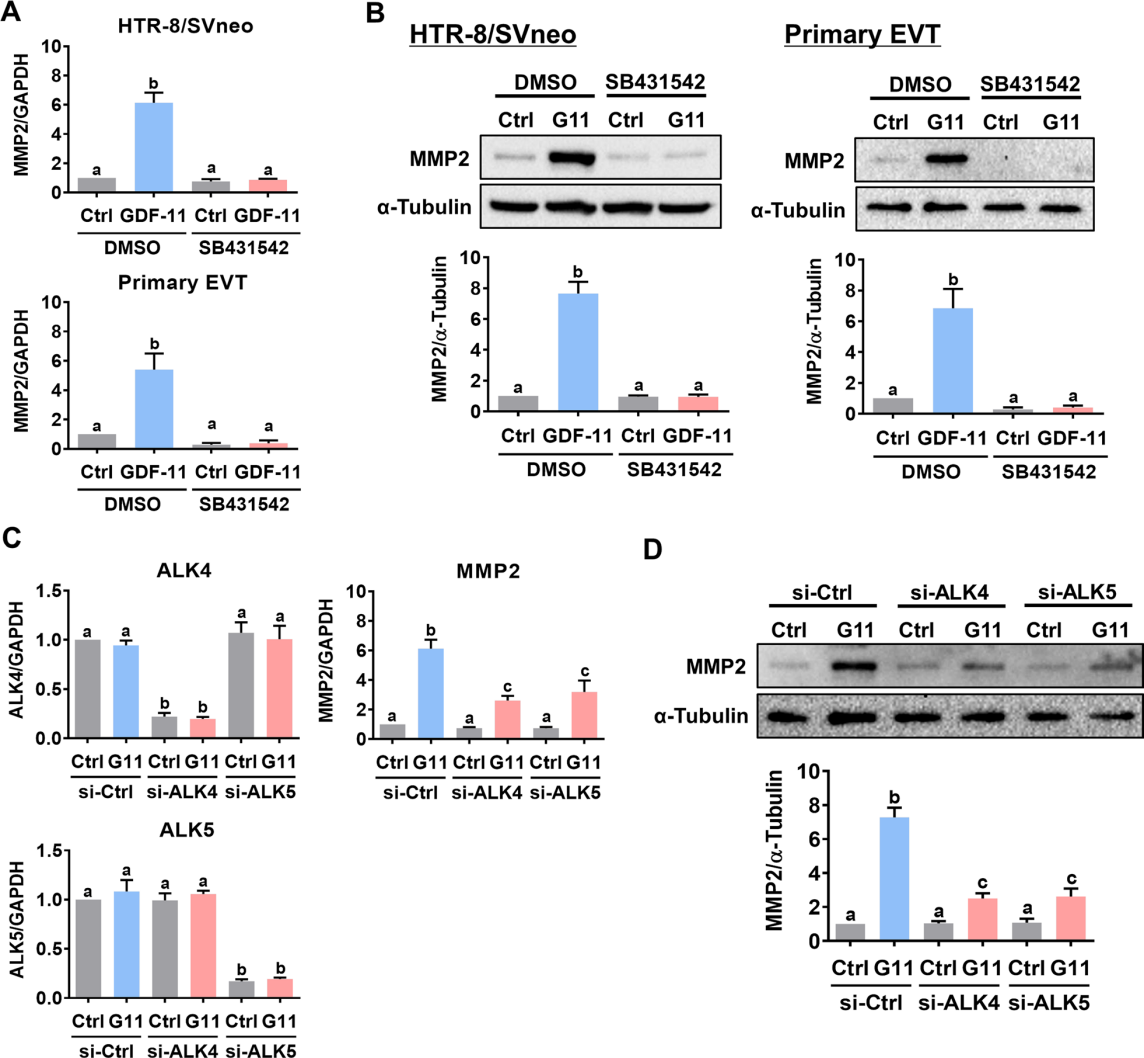
ALK4 and ALK5 mediate the stimulatory effect of GDF-11 on MMP2 expression in human EVT cells. **A** and **B**, HTR-8/SVneo and primary EVT cells were pretreated with vehicle control (DMSO) or 10 µM SB431542 for 1 h, and then treated with 30 ng/mL GDF-11 (G11) for 24 h. The mRNA (**A**) and protein (**B**) levels of MMP2 were examined by RT-qPCR and western blot, respectively. **C** and **D**, HTR-8/SVneo cells were transfected with 50 nM control siRNA (si-Ctrl), ALK4 siRNA (si-ALK4), or ALK5 siRNA (si-ALK5) for 48 h, and then treated with 30 ng/mL GDF-11 (G11) for 24 h. The ALK4, ALK5, MMP2 mRNA (**C**), and MMP2 protein (**D**) levels were examined by RT-qPCR and western blot, respectively. The results are expressed as the mean ± SEM of at least three independent experiments. Multiple comparisons were analyzed using one-way ANOVA followed by Tukey’s multiple comparison test. Values that are statistically different from one another (*p* < 0.05) are indicated by different letters. The values with any common letter are not significantly different

In a context-dependent manner, ALK4, ALK5, and/or ALK7 are required for the biological function of GDF-11. Pretreatment of HTR-8/SVneo and primary EVT cells with SB431542, a potent ALK4/5/7 inhibitor [26], blocked the stimulatory effects of GDF-11 on the MMP2 mRNA and protein levels (Fig. 2A, B). It has been shown that, in humans, ALK7 is mainly expressed in the adipose tissue and the expression levels of ALK7 are very low in the placenta [27]. Because SB431542 inhibits the function of ALK4 and ALK5, to further distinguish the involvement of ALK4 and ALK5 in GDF-11-induced upregulation of MMP2 expression, the siRNA-mediated knockdown approach was applied to block the function of ALK4 and ALK5 specifically in HTR-8/SVneo cells. As shown in Fig. 2C, ALK4 siRNA specifically downregulated the endogenous ALK4 mRNA levels without affecting the endogenous ALK5 mRNA levels and vice versa for ALK5 siRNA. The stimulatory effect of GDF-11 on MMP2 mRNA levels was attenuated by the knockdown of ALK4 or ALK5. Western blot analysis showed similar results that both ALK4 and ALK5 were involved in the GDF-11-induced upregulation of MMP2 protein levels in HTR-8/SVneo cells (Fig. 2D).

### GDF-11 upregulates MMP2 expression by activating SMAD2/3 signaling pathways

**Fig. 3:**
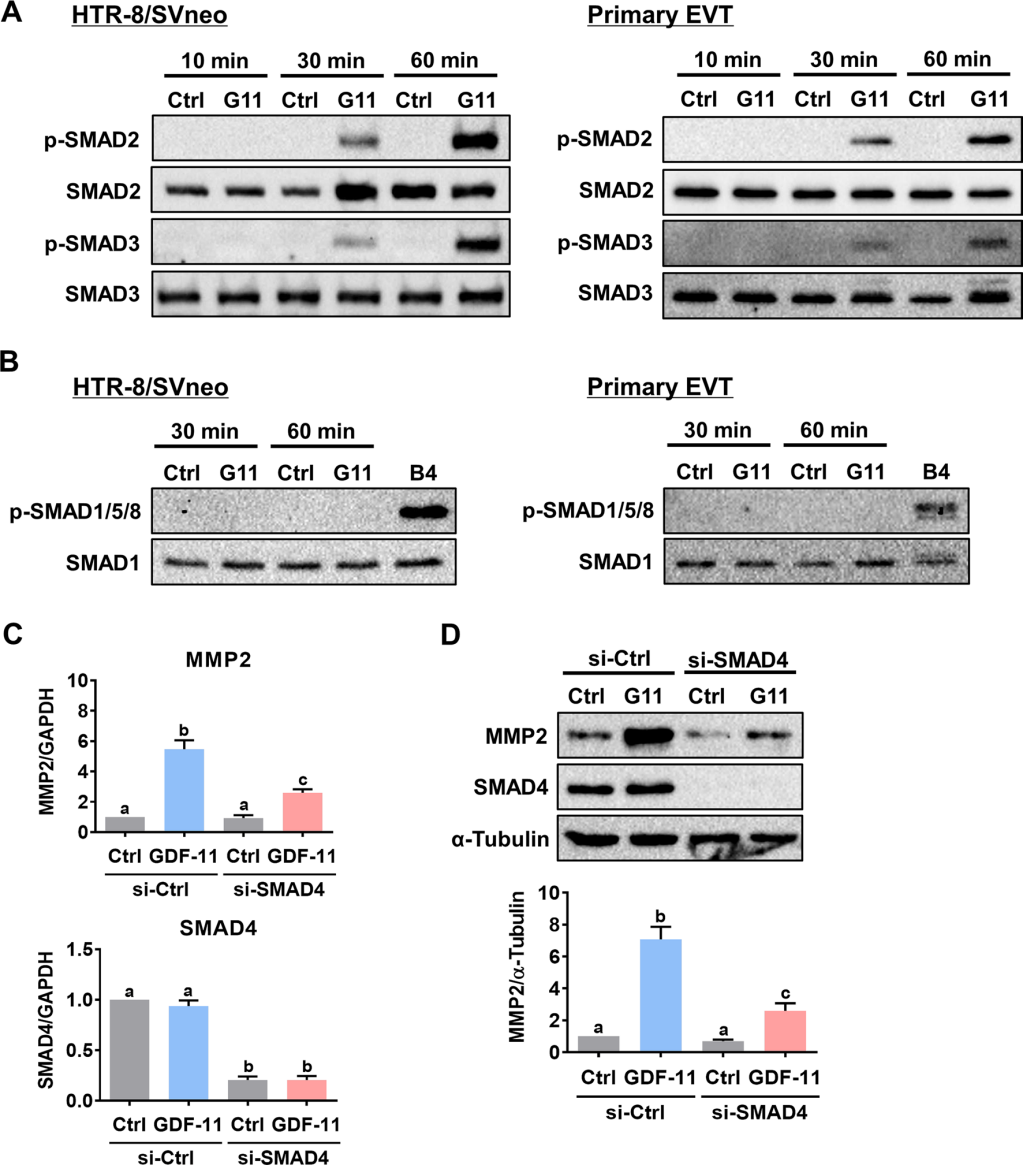
GDF-11-activated SMAD2/3 signaling pathways are required for GDF-11-induced MMP2 expression in human EVT cells. **A**, HTR-8/SVneo and primary EVT cells were treated with 30 ng/mL GDF-11 (G11) for 10, 30, and 60 min. The levels of phosphorylated and total SMAD2 and SMAD3 were determined by western blot. **B**, HTR-8/SVneo and primary EVT cells were treated with 30 ng/mL GDF-11 (G11) for 30 and 60 min. The levels of the phosphorylated SMAD1/5/8 and total SMAD1 were determined by western blot. Treatment of cells with 10 ng/mL BMP-4 (B4) for 60 min was used as a positive control for the phosphorylation of SMAD1/5/8. **C** and **D**, HTR-8/SVneo cells were transfected with 50 nM control siRNA (si-Ctrl) or SMAD4 siRNA (si-SMAD4) for 48 h, and then treated with 30 ng/mL GDF-11 (G11) for 24 h. The mRNA (**C**) and protein (**D**) levels of MMP2 and SMAD4 were examined by RT-qPCR and western blot, respectively. The results are expressed as the mean ± SEM of at least three independent experiments. Multiple comparisons were analyzed using one-way ANOVA followed by Tukey’s multiple comparison test. Values that are statistically different from one another (*p* < 0.05) are indicated by different letters. The values with any common letter are not significantly different

**Fig. 4:**
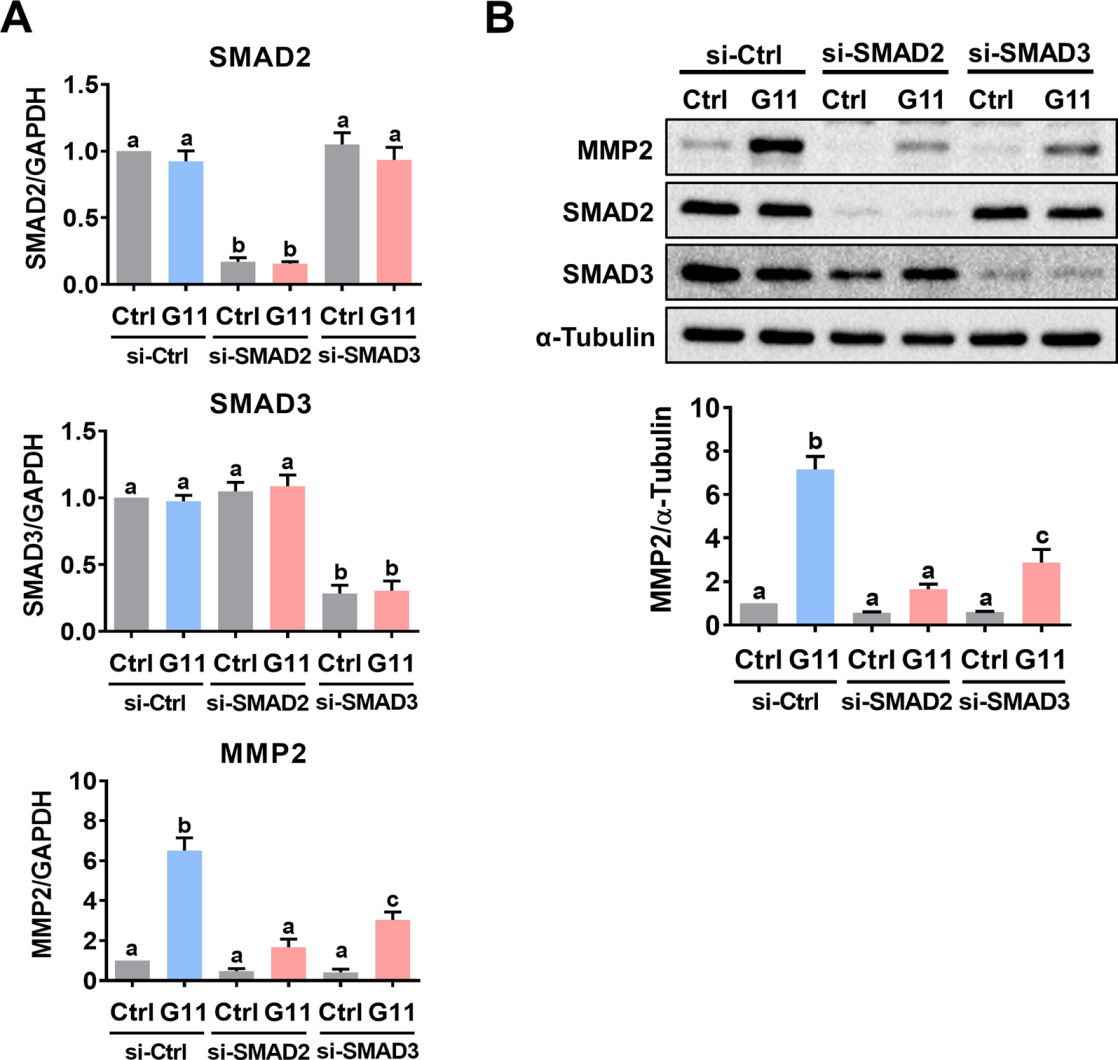
Both SMAD2 and SMAD3 participate in the GDF-11-induced MMP2 expression in HTR-8/SVneo cells. **A** and **B**, Cells were transfected with 50 nM control siRNA (si-Ctrl), SMAD2 siRNA (si-SMAD2), or SMAD3 siRNA (si-SMAD3) for 48 h, and then treated with 30 ng/mL GDF-11 (G11) for 24 h. The mRNA (**A**) and protein (**B**) levels of MMP2, SMAD2, and SMAD3 were examined by RT-qPCR and western blot, respectively. The results are expressed as the mean ± SEM of at least three independent experiments. Multiple comparisons were analyzed using one-way ANOVA followed by Tukey’s multiple comparison test. Values that are statistically different from one another (*p* < 0.05) are indicated by different letters. The values with any common letter are not significantly different

In different cell types, GDF-11 activates SMAD2/3 and SMAD1/5/8 signaling pathways. Treatment of HTR-8/SVneo and primary EVT cells with GDF-11 induced the phosphorylation levels of SMAD2 and SMAD3 indicating their activations. However, the SMAD1/5/8 signaling pathways were not activated by the GDF-11 in both HTR-8/SVneo and primary EVT cells. We used BMP4 as the positive control for the activation of the SMAD1/5/8 signaling pathways (Fig. 3A,B). To define the involvement of SMAD signaling pathways in GDF-11-induced upregulation of MMP2 expression, the common SMAD for the functional SMAD signaling pathways, SMAD4, was knocked down by the specific siRNA. As shown in Fig. 3C, D, the knockdown of SMAD4 attenuated the stimulatory effects of GDF-11 on both MMP2 mRNA and protein levels in HTR-8/SVneo cells. Although in most contexts, the function of SMAD2 and SMAD3 are indistinguishable, these two similar intracellular signaling proteins can exert distinct roles under some conditions [28]. To further define the individual role of SMAD2 and SMAD3 in mediating the stimulatory effect of GDF-11 on MMP2 expression, the expression of endogenous SMAD2 or SMAD3 was knocked down by the specific siRNA. Transfection of HTR-8/SVneo cells with SMAD2 siRNA specifically downregulated the endogenous SMAD2 mRNA levels without affecting the SMAD3 mRNA levels and vice versa for the SMAD3 siRNA transfection. Knockdown of SMAD2 or SMAD3 attenuated the stimulatory effect of GDF-11 on MMP2 mRNA levels (Fig. 4A). Western blot results showed similar results that both SMAD2 and SMAD3 were required for the GDF-11-induced upregulation of MMP2 expression in HTR-8/SVneo cells (Fig. 4B).

### ID2 mediates GDF-11-induced upregulation of MMP2 expression

**Fig. 5:**
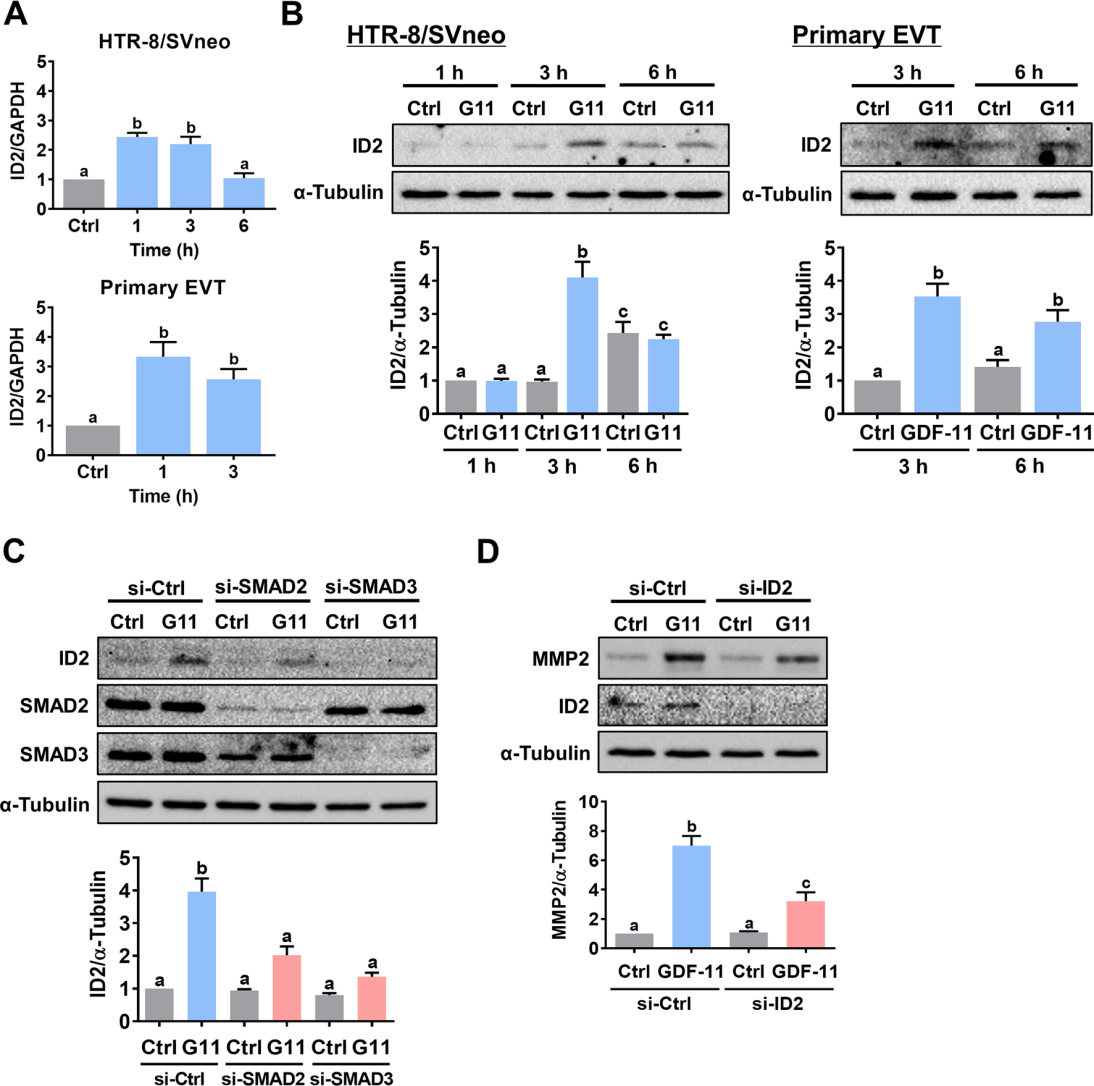
Induction of ID2 is required for the GDF-11-induced MMP2 expression in human EVT cells. **A** and **B**, HTR-8/SVneo and primary EVT cells were treated with 30 ng/mL GDF-11 (G11) for 1, 3, and 6 h. The mRNA (**A**) and protein (**B**) levels of ID2 were examined by RT-qPCR and western blot, respectively. **C**, HTR-8/SVneo cells were transfected with 50 nM control siRNA (si-Ctrl), SMAD2 siRNA (si-SMAD2), or SMAD3 siRNA (si-SMAD3) for 48 h, and then treated with 30 ng/mL GDF-11 (G11) for 3 h. The protein levels of ID2, SMAD2, and SMAD3 were examined by western blot. **D**, Cells were transfected with 50 nM control siRNA (si-Ctrl) or ID2 siRNA (si-ID2) for 48 h, and then treated with 30 ng/mL GDF-11 (G11) for 24 h. The protein levels of MMP2 and ID2 were examined by western blot. The results are expressed as the mean ± SEM of at least three independent experiments. Multiple comparisons were analyzed using one-way ANOVA followed by Tukey’s multiple comparison test. Values that are statistically different from one another (*p* < 0.05) are indicated by different letters. The values with any common letter are not significantly different

In mammals, four inhibitor of DNA-binding (ID) proteins, ID1-4, that share extensive sequence homology in the HLH motif have been identified. ID proteins can be regulated by the TGF-β superfamily [29]. ID2 has been shown to mediate the expression of MMP2 in cancer cells [30, 31]. Therefore, we examined whether ID2 is involved in the GDF-11-induced upregulation of MMP2 expression. Treatment with GDF-11 induced ID2 mRNA and protein levels in both HTR-8/SVneo and primary EVT cells (Fig. 5A, B). In HTR-8/SVneo cells, knockdown of SMAD2 or SMAD3 abolished the stimulatory effect of GDF-11 on ID2 protein levels (Fig. 5C). Importantly, siRNA-mediated knockdown of ID2 attenuated the GDF-11-induced MMP2 protein levels (Fig. 5D). Collectively, these results indicate that GDF-11-induced ID2 is required for the induction of MMP2 expression in human EVT cells.

### ID2-mediated MMP2 expression is required for GDF-11-stimulated EVT cell invasion

**Fig. 6:**
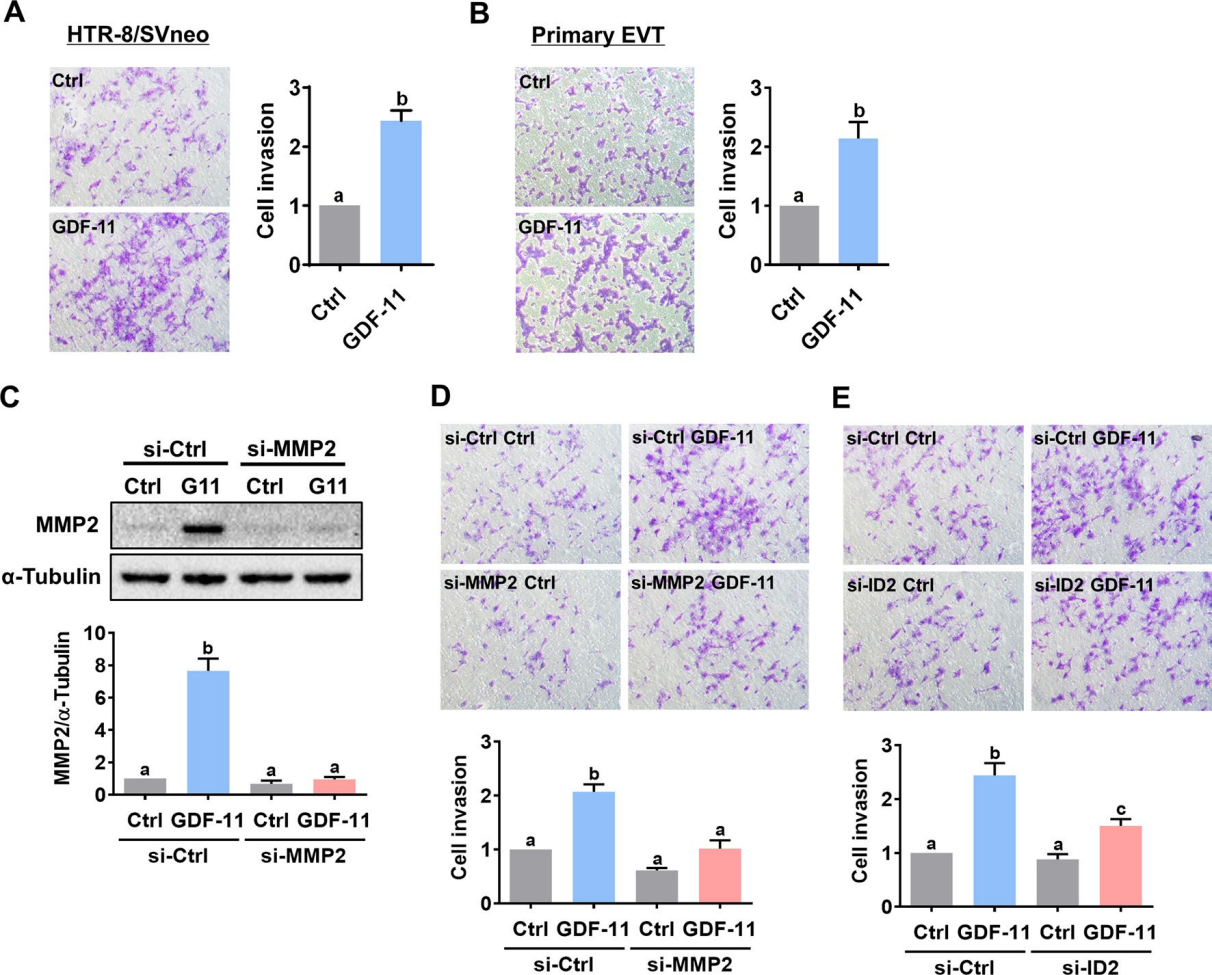
ID2-mediated MMP2 expression is required for the GDF-11-stimulated human EVT cell invasion. **A** and **B**, HTR-8/SVneo (**A**) and primary EVT (**B**) cells were treated with 30 ng/mL GDF-11 and the cell invasiveness was measured by the Matrigel transwell invasion assay. **C**, HTR-8/SVneo cells were transfected with 50 nM control siRNA (si-Ctrl) or MMP2 siRNA (si-MMP2) for 48 h, and then treated with 30 ng/mL GDF-11 (G11) for 24 h. The protein levels of MMP2 were examined by western blot. **D** and **E**, HTR-8/SVneo cells were transfected with 50 nM control siRNA (si-Ctrl), MMP2 siRNA (si-MMP2) (**D**), or ID2 siRNA (si-ID2) (**E**) for 48 h, and then treated with 30 ng/mL GDF-11 (G11). The cell invasiveness was measured by the Matrigel transwell invasion assay. For the invasion assay, the top panel shows representative photos of the invaded cells. The bottom panels show summarized quantitative results. The results are expressed as the mean ± SEM of at least three independent experiments. Multiple comparisons were analyzed using one-way ANOVA followed by Tukey’s multiple comparison test. Values that are statistically different from one another (*p* < 0.05) are indicated by different letters. The values with any common letter are not significantly different

To examine the effect of GDF-11 on the invasiveness of EVT cells, the Matrigel-coated transwell invasion assay was applied. Treatment with GDF-11 stimulated invasiveness in both HTR-8/SVneo and primary EVT cells (Fig. 6A, B). To define the role of MMP2 in GDF-11-stimulated cell invasion, the function of MMP2 was blocked by the siRNA-mediated knockdown approach in HTR-8/SVneo. As shown in Fig. 6C, both endogenous and the GDF-11 upregulated MMP2 protein levels were downregulated by the MMP2 siRNA. In addition, the stimulatory effect of GDF-11 on HTR-8/SVneo cell invasiveness was attenuated by the siRNA-mediated knockdown of MMP2 (Fig. 6D). Moreover, the knockdown of ID2 also attenuated the GDF-11-induced HTR-8/SVneo cell invasion (Fig. 6E). Taken together, these results indicate that the ID2-mediated induction of MMP2 expression is required for the GDF-11-stimulated human EVT cell invasion.

### Discussion

Previous immunohistochemical analysis results have shown that GDF-11 is expressed in the placenta and the secretory phase of the human endometrium which suggests the role of GDF-11 in the regulation of implantation [32]. However, thus far, there is no study directly investigating the biological role of GDF-11 in the human placenta or endometrium. In the present study, we revealed the novel biological role of GDF-11 in stimulating the human placental EVT cell invasion. The stimulatory effect of GDF-11 on invasiveness was mediated by the induction of MMP2. Our results also delineated that ALK4 and ALK5-mediated SMAD2/3 signaling pathways were involved in the GDF-11-induced MMP2 expression. In addition, we showed that the transcriptional regulator ID2 mediated the MMP2 expression induced by the GDF-11. This study suggests that GDF-11 may play an important role in the implantation by regulating EVT cell invasion.

To date, only a handful of studies have investigated the roles of GDF-11 on cell migration/invasion. However, most studies are done on cancer cells. In oral cancer cells, treatment with GDF-11 stimulates cell migration and induced expressions of MMP2 and MMP9 [33]. In contrast, GDF-11 suppresses invasiveness in human liver, pancreatic, and triple-negative breast cancer cells [34–36]. These results suggest the effect of GDF-11 on cell migration/invasion is in a cell-type-dependent manner. GDF-11 has remarkable sequence similarity with GDF-8 [13]. Our recent study shows that GDF-8 stimulates HTR-8/SVneo cell invasion by upregulating MMP2 but not MMP9 expression [21]. In the present study, we observed that GDF-11 stimulated MMP2 but not MMP9 expressions which are the same as the results obtained from GDF-8. It is known that the biological function of GDF-8 in myogenic cells is mainly mediated by ALK4, while ALK5 mediates GDF-8 function in non-myogenic cells [37]. Agreeing with this, our previous study shows that the stimulatory effect of GDF-8 on MMP2 expression in HTR-8/SVneo cells is mediated by ALK5 but not ALK4 [21]. Interestingly, in the same cells, here we revealed that both ALK4 and ALK5 were involved in the GDF-11-induced upregulation of MMP2. Collectively, these results indicate that GDF-11 and GDF-8 utilize different receptors to exert the same pro-invasive role in human EVT cells.

It has been shown that GDF-11 and GDF-8 predominantly utilize ALK4 or ALK5 to elicit signal transduction via SMAD2 and SMAD3 [22]. Using the siRNA-mediated knockdown approach, we defined that both SMAD2 and SMAD3 were involved in GDF-11-induced MMP2 expression in HTR-8/SVneo cells. GDF-11 is also known as BMP-11. Like other BMP proteins, one study has demonstrated that GDF-11 can activate SMAD1/5/8 signaling pathways in human umbilical vein endothelial cells [38, 39]. Using BMP4 as a positive control, we found that SMAD1/5/8 signaling pathways were not activated by the GDF-11 in both HTR-8/SVneo and primary EVT cells. It is interesting to note that inhibiting SMAD signaling pathways did not fully block the stimulatory effect of GDF-11 on MMP2 expression. These results suggest the involvement of other SMAD-independent non-canonical signaling pathways in GDF-11-stimulated MMP2 expression. Thus, further investigation is warranted to explore whether other signaling pathways contribute to GDF-11-induced MMP2 expression in human EVT cells.

Because lacking the DNA-binding domain, ID proteins act as transcriptional modulators by heterodimerizing with bHLH transcription factors to inhibit their DNA binding activity [40, 41]. The expression of MMP2 is regulated by the ID2 and ID2 promotes cell migration and invasion in different types of human cancer cells [30, 31, 42]. Consistent with those previous studies, using ID2 siRNA, we showed that induction of ID2 was required for the GDF-11-stimulated MMP2 expression. In addition, the knockdown of ID2 attenuated the GDF-11-induced invasiveness in HTR-8/SVneo cells. In the human placenta, after differentiation, both mRNA and protein levels of ID2 are downregulated in cytotrophoblast cells. Overexpression of ID2 inhibits cytotrophoblast cell invasion but dramatically promotes cell migration [43]. The cause of the opposite effects on cell migration and invasion by ID2 overexpression in human cytotrophoblast cells remains unknown. However, the anti-invasive effect of ID2 in human cytotrophoblast cells is in contrast to our results in HTR-8/SVneo cells. The major factor that leads to this distinct effect may be the supra-physiological overexpression which can commonly induce off-target effects. In addition, the HTR-8/SVneo cell line was generated using first-trimester EVT cells infected with SV40 large T antigen [24]. The immortalization process and the different natures of cytotrophoblast and EVT cells could also result in the opposite effects of ID2 in trophoblast cell invasion.

## Conclusions

In summary, in the present study, we report a novel function of GDF-11 in the human placenta which GDF-11 stimulates human EVT cell invasion by upregulating MMP2 expression. Mechanically, we delineate that ALK4/5-mediated SMAD2/3 signaling pathways are involved in the stimulatory effect of GDF-11 on MMP2 expression. In addition, we show that ID2 protein is upregulated by GDF-11 and that is required for GDF-11-stimulated MMP2 expression and EVT cell invasion. Our study not only discovers the function of GDF-11 but also provides important insights into the regulation of MMP2 expression in the human placenta.

## Supplementary Information

**Additional file 1: Fig. S1.** The isolation and characterization of human EVT cells. A, The representative photos for the outgrowth of human EVT cells from a villous explant (upper panel) and the morphology of isolated EVT cells (lower panel). B, The expressions of cytokeratin-7, HLA-G, and vimentin were examined by immunofluorescence staining. C, The expressions of cytokeratin-7, HLA-G, and vimentin were examined by western blot. SKOV3 human ovarian cancer cells were used as positive controls for the expression of cytokeratin-7 and vimentin.

## Data Availability

The data that support the findings of this study are available from the corresponding author upon reasonable request.

## Abbreviations

ALK: Activin receptor-like kinase
BMP: Bone morphogenetic protein
EVT: Extravillous trophoblast
GDF: Growth differentiation factor
ID: Inhibitor of DNA-binding protein
MMP: Matrix metalloproteinase
TGF-β: Transforming growth factor-beta
