# Supplementary figures and images for: GDF-11 promotes human trophoblast cell invasion by increasing ID2-mediated MMP2 expression
Source: Cell Commun Signal. 2022 Jun 15;20:89. doi: 10.1186/s12964-022-00899-z (PMC9202197; doi:10.1186/s12964-022-00899-z)

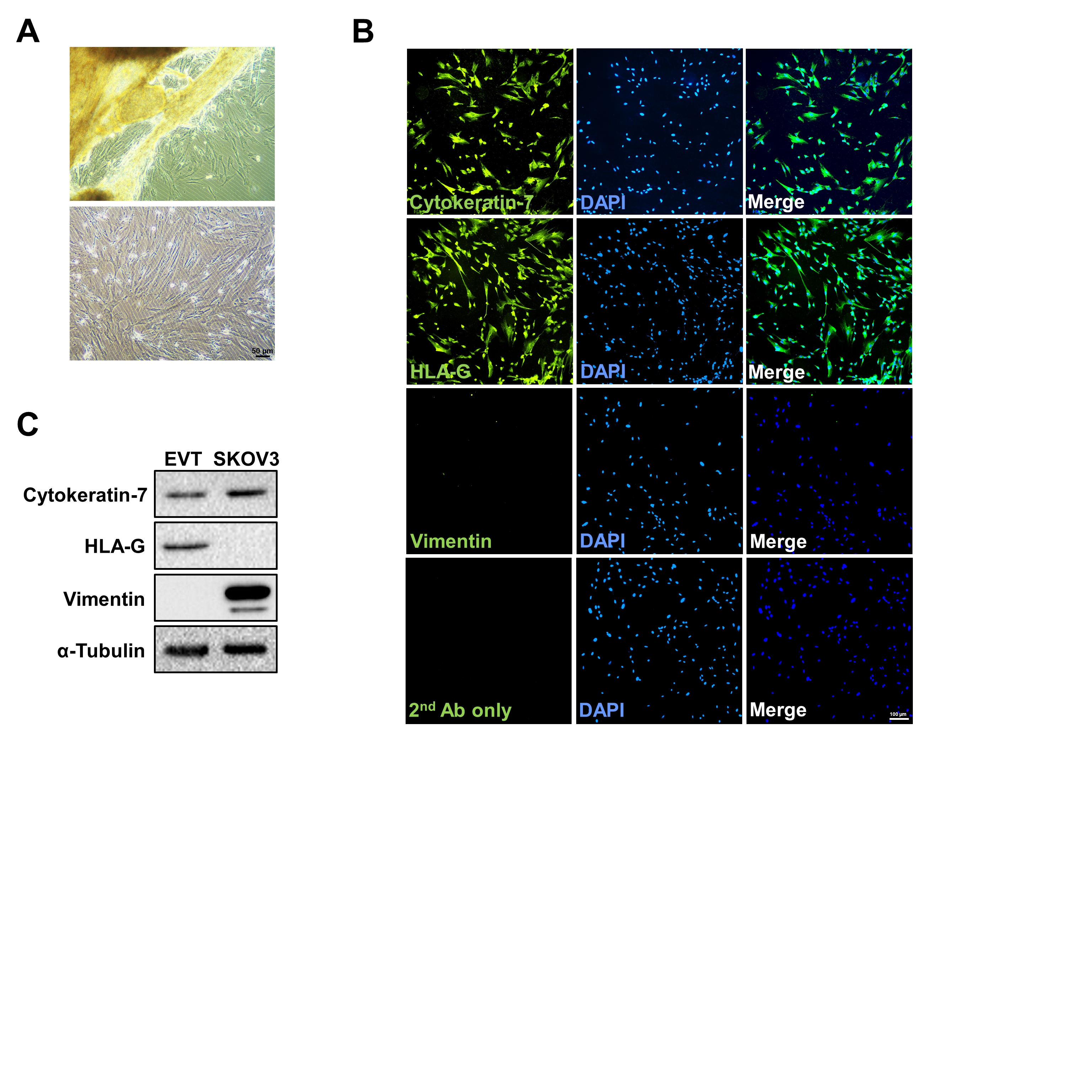

Supplement: Supplementary file 2 — Additional file 1: Fig. S1. The isolation and characterization of human EVT cells. A, The representative photos for the outgrowth of human EVT cells from a villous explant (upper panel) and the morphology of isolated EVT cells (lower panel). B, The expressions of cytokeratin-7, HLA-G, and vimentin were examined by immunofluorescence staining. C, The expressions of cytokeratin-7, HLA-G, and vimentin were examined by western blot. SKOV3 human ovarian cancer cells were used as positive controls for the expression of cytokeratin-7 and vimentin. [file 12964_2022_899_MOESM2_ESM.tif]
